# A latent class analysis of biosecurity attitudes and decision-making strategies of swine producers in the United States

**DOI:** 10.1038/s41598-024-67385-z

**Published:** 2024-08-05

**Authors:** Richmond Silvanus Baye, Asim Zia, Scott C. Merrill, Eric M. Clark, Julia M. Smith, Christopher Koliba

**Affiliations:** 1 Department of Community Development and Applied Economics, University of Vermont, Burlington, VT USA; 2 Social-Ecological Gaming and Simulation Lab, University of Vermont, Burlington, VT USA; 3 Gund Institute for Environment, University of Vermont, Burlington, VT USA; 4 Department of Plant and Soil Science, University of Vermont, Burlington, VT USA; 5 Department of Animal and Veterinary Sciences, University of Vermont, Burlington, VT USA; 6 School of Public Affairs and Administration, University of Kansas, Lawrence, KS USA; 7 Vermont Complex Systems Center, University of Vermont, Burlington, VT USA

**Keywords:** African swine fever, Biosecurity behavior, Latent class analysis, Risk factors, Socioeconomic scenarios, Health policy

## Abstract

The 2018 African swine fever (ASF) outbreak highlighted the importance of biosecurity in food production systems. Despite the significant economic impacts, the sociopsychological consequences on decision-making have been overlooked. Previous studies have focused on algebraic models and simulation-based models without considering the complex psychological and social factors that influence farmers' biosecurity behaviors and decision-making processes. This study aims to classify livestock producers into distinct subgroups based on their attitudes towards biosecurity. We conducted a survey presenting producers with three scenarios to assess their willingness to report suspected ASF cases, trust in government agencies, risk perception, biosecurity knowledge, willingness to purchase livestock insurance, motivation to invest in biosecurity, readiness to report suspected infections, and intention to contact a veterinarian. Using latent class analysis, we identified three distinct classes: Biosecurity Sceptics, Biosecurity Compliant, and Biosecurity Ultra-Compliant. Our results show that producer characteristics significantly influence biosecurity attitudes and class membership, with small-scale producers less likely to adopt ultra-compliant biosecurity practices. Attending at least one eradication program encouraged biosecurity compliance. This research informs the design of targeted food policy and risk communication strategies that account for attitudes of livestock producers to encourage biosecurity adoption and reduce the likelihood of Tier 1 disease incursion.

## Introduction

The threat of global pandemics affecting the world’s livestock is very real. The 2018 African swine fever (ASF) epidemic in China, which resulted in the culling of over 40 million pigs, has far-reaching implications across multiple scales and in different countries. This underscores the criticality of investing in biosecurity measures to prevent the spread of transboundary animal diseases (TADs)^1,2^. The outbreak led to significant culling of animals, impacting food security, trade dynamics, global animal health, and the environment^3^. Vietnam experienced the culling of approximately six million pigs between 2019 and 2021, resulting in a severe decline in pork production, a reduction in exports, and adverse effects on the country's gross domestic product (GDP)^4^. Beyond the immediate economic impacts that focus on how individuals respond to disease risk outbreaks and how decisions vary across different circumstances^5,6^, there are socio-psychological factors that linger and have a lasting impact on the decision-making process of producers’ post-outbreak^7,8^. Such events highlight the urgent need to understand producers' biosecurity attitudes and behaviors to reduce the likelihood of similar incursions.

According to Jones et al.^9^, an accidental introduction of ASF will have dire ramifications on multiple domains with impacts on social, economic, environmental, and psychological perspectives. Carriquiry et al.^10^ demonstrate that a hypothetical ASF outbreak in the United States (US) could result in substantial declines in pork production, extensive job losses, and significant revenue losses over a decade. The response measures implemented in the event of an outbreak in the United States, such as lockdowns, active surveillance, depopulation, and export controls, will contribute to these losses^8,11^. Furthermore, the indemnity payment program, intended to encourage producers to report suspected cases and adopt biosecurity practices, raises challenges and potential conflicts in producers' motivations and decision-making^12–16^.

At the frontlines, several studies show that biosecurity behavior demonstrated by producers may be influenced by the producers’ knowledge of biosecurity, willingness to buy livestock insurance, and their readiness to report infections on their farms^17–19^. Knowledge on biosecurity is essential for disease prevention, and lack of knowledge together with low-risk perception and low levels of trust in government officials to effectively manage an outbreak may inhibit biosecurity adoption^17,18, 20^. Evidence from Elbers et al.^21^ revealed that Dutch swine producers had a rather limited knowledge of the clinical signs of classical swine fever, and famers placed the responsibility of judging a clinical suspect completely in the hands of the veterinarian. Moreover, studies have also shown that the source of information plays a critical role in the way producers respond to biosecurity^22^. For instance, Haffernan et al. (2008) found that a neutral source of information such as farmers media or journal was more trusted and relied upon than government-led information sources. They observed that producers had a strong negative attitude towards specific sources of biosecurity information such as government leaflets.

Similar to government-sponsored indemnification programs, livestock insurance offers an opportunity to prevent foreign animal diseases. However, the challenges of moral hazard and adverse selection can affect a farmer's production decisions and potentially undermine the effectiveness of a livestock insurance program. For instance, Mato-Amboage et al.,^23^ found that farmers in Spain were less likely to buy livestock insurance if they were required to have already implemented certain biosecurity measures in order to receive indemnity if they made a claim. While this is the case, alternative risk management strategies are available, including cost-sharing arrangements between private and public sectors, co-payments and contract farming have been shown to mitigating these challenges. Hennessy^24^ and Beckie et al.^25^, for example, proposed insurance designs where those who comply with certain biosecurity management practices are entitled to receive linked reductions in premiums, or government compensation beyond a certain minimum level. Gramig et al.^26^ show that if a livestock farmer is priced below market value, they may purchase more insurance to cover a larger portion of their livestock. However, if they are priced above the market value, they may be disincentivized to invest in biosecurity. This source of information asymmetry poses challenges for developing and implementing an effective biosecurity strategy to prevent outbreaks of TADs.

Several previous studies have sought to understand the interactions of indemnity payments, biosecurity adoption, disease reporting, livestock insurance, risk perception, trust in government agencies, and inherent beliefs^12,18, 26, 27^ and have shown potential gains from policies that tie indemnity payments to biosecurity efforts^28^. Sumner et al.^29^ observe that producers’ perceptions and attitudes can vary significantly, which can be attributed to the inherent public good characteristics of infectious animal disease and the associated external costs and benefits. Our study is motivated by the need to prevent the introduction of ASF into the US. As such, we focus on understanding behavioral factor and attitudes that drive these preventative decisions on their farms. Following Otieno et al.^30^, our research is grounded in the assumption that preventative measures are more efficient and cost-effective than curative driven measures. Apart from the prevention of an outbreak, these measures have been shown to boost the immune response of animals, improve animal welfare and improve profit margins^31,32^.

Although adopting biosecurity practices exists among producers, it varies by the degree of investments and even in some situations, non-existent due to cost considerations and responses to public policy. In practice, farmers often implement biosecurity measures selectively or partially, adopting some while neglecting others. While existing studies have demonstrated the potential benefits of biosecurity adoption and the factors driving decisions to implement biosecurity measures on farms, most have not explored the underlying behaviors, attitudes, and perceptions influencing these decisions, either under the status quo or in response to changes in policy (such as indemnity).

The problem is most of these studies^12,33, 34^ rely on algebraic models or simulation models to understand disease epidemiology without considering the role of human behavior. Very few studies including^6,20, 27, 30, 35, 36^ have applied socio-psychological approaches and gaming data to understand these decisions. For instance, in identifying the determinants of farmers’ biosecurity attitudes, Richens et al.^36^ observed that a lack of biosecurity awareness was not the issue but the fact that farmers perceived the inevitable outbreak of disease to be out of their control. On the contrary, Toma et al.^37^ observed that farmers perceived importance of specific biosecurity strategies and perceived usefulness of biosecurity information sources influenced their decision to apply biosecurity measures. These findings suggest that understanding human behavior and socio-psychological factors play a significant role in capturing biosecurity decisions.

In light of the paucity in scientific literature, the present study aims to understand patterns of behavior, perceptions, beliefs, and attitudes to biosecurity adoption in response to a policy change on indemnity. To understand these complex factors influencing their attitudes and decisions towards the uptake ex-ante biosecurity practices on their farms, we employ a latent class analysis (LCA) to a survey data aimed at identifying unobserved homogeneous subgroups of producers representing their biosecurity attitudes and decisions making strategies. We also estimate the “one-step” latent class regression to predict the potential drivers of the unobserved classes of biosecurity behaviors.

By using a latent class approach, we aim to manifest the latent phenomenon underlying producers' preferences, attitudes, and behaviors. This approach will enable an exploration of the complementarities between biosecurity investment, livestock insurance purchase, and disease reporting, ultimately leading to a more comprehensive understanding of producers' decision-making processes. This will supplement existing behavioral and sociological literature that used nudges or nonfinancial incentives to motivate biosecurity adoption and encourage disease reporting^20,21^. The study further implements a latent class regression in which the probability of latent class membership is predicted by a set of covariates. This approach allows us to reduce bias while allowing the individuals priors to vary depending on their observed covariate^38^.

The proposed research contributes to the literature by offering a novel perspective on the motivations behind producers' choices regarding biosecurity attitudes. It also addresses the need for targeted food policy and associated risk communication strategies for designing interventions by identifying distinct classes of producers with varying attitudes and behaviors. By understanding these unobserved factors and grouping producers accordingly, policymakers can design more effective and tailored interventions to combat TADs such as ASF.

## Results and discussion

### Descriptive statistics

With 422 responses, the survey aimed to understand the underlying behavioral attitudes of swine producers relating to their intent to adopt preventive practices on their farms. We present the frequency distribution of responses in Table 1. Each variable was measured on a Likert scale ranging from 1 to 5, where 5 indicated strong agreement, 3 indicated unsure, and 1 indicated strong disagreement.

**Table 1:** Frequency table of the selected variables in the model.

Variable	Category	Count	Percentage
Intention to call a veterinarian to examine their herd	Strongly disagree	5	1.18%
	Disagree	9	2.13%
	Unsure	39	9.24%
	Agree	257	60.90%
	Strongly agree	112	26.54%
	Total		422
Motivation to self-invest in biosecurity	Strongly disagree	2	0.47%
	Disagree	19	4.50%
	Unsure	62	14.69%
	Agree	237	56.16%
	Strongly agree	102	24.17%
	Total		422
Likelihood of buying livestock insurance	Strongly disagree	2	0.47%
	Disagree	27	6.40%
	Unsure	91	21.56%
	Agree	188	44.55%
	Strongly agree	114	27.01%
	Total		422
Readiness to report infections	Strongly disagree	3	0.71%
	Disagree	6	1.42%
	Unsure	53	12.56%
	Agree	207	49.05%
	Strongly agree	153	36.26%
	Total		422
Risk perception	Strongly disagree	18	4.27%
	Disagree	43	10.19%
	Unsure	111	26.30%
	Agree	191	45.26%
	Strongly agree	59	13.98%
	Total		422
Trust in government agencies	Strongly disagree	11	2.61%
	Disagree	30	7.11%
	Unsure	131	31.04%
	Agree	178	42.18%
	Strongly agree	72	17.06%
	Total	422	100.00%
Confidence in neighbor to biosecure	Strongly disagree	16	3.79%
	Disagree	39	9.24%
	Unsure	111	26.30%
	Agree	202	47.87%
	Strongly agree	54	12.80%
	Total	422	100.00%
Gender	Female	152	36.02%
	Male	270	63.98%
	Total		422
Farm size	Large	52	12.38%
	Medium	30	7.14%
	Small	338	80.48%
	Total		420
Participating in eradication program	At least one eradication program	356	84.36%
	None	66	15.64%
	Total		422
Income shares from production	Small income share	113	26.78%
	Medium income share	125	29.62%
	High income share	184	43.60%
	Total		422

Starting with the motivation to self-invest in biosecurity, the likelihood of buying livestock insurance, and the readiness to report suspected infected livestock on their farms, the respondents were presented with a preamble and were asked whether they agreed or disagreed with the statement: “If funds were available from the government but were contingent on showing your biosecurity plans.” “*It will motivate me to invest and show my biosecurity plans.*” Results from the survey showed that about 23% of the respondents strongly agreed that they would self-invest in biosecurity practices, with 56% also agreeing to the statement and 15% expressing uncertainty; only 5% disagreed, and 1% strongly disagreed.

Regarding buying livestock insurance, 27% strongly agreed, while 44% agreed. However, 22% expressed uncertainty, and 6% and 1% disagreed and strongly disagreed, respectively. Approximately 35% of respondents strongly agreed to promptly report suspected cases of infected livestock on their farms, 49% agreed, and 13% were unsure. Two percent (2%) of the respondents disagreed, and approximately 1% strongly disagreed. With reference to the intention to call a veterinarian for their herd examination, 25% strongly agreed, with an additional 61% agreeing, 10% were uncertain, while 2% and 2% disagreed or strongly disagreed, respectively.

The respondents were also asked to rate their level of trust in government agencies to manage an outbreak. Approximately 17% of producers strongly agreed that they trust government agencies to effectively manage disease outbreaks, about 42% of them agreed that they trust government to effectively manage an outbreak**.** While roughly 30% were unsure about government agency’s ability to manage disease. Approximately 8% disagreed, while 3% strongly disagreed. The producers were also presented with a normative question to assess a specific attitude about their biosecurity practices on their farms. We call this “farmer knowledge of biosecurity.” Approximately 22% strongly agreed that workers and visitors should wear appropriate gear during farm visits, followed by approximately 53% agreeing that workers and visitors should wear appropriate gear during farm visits. While 19% were uncertain, and approximately 6% disagreed. We also asked questions about their risk-perception. About 14% strongly agree, 45% agree, while 31% are unsure that that their operations are at risk of ASF.

We recoded the share of income derived from production into three groups: low (0–30%), medium (40–60%), and high (70–100%). Overall, most of the farmers belonged to the high share of income category. We also asked questions about producers attending at least one eradication program and about 84% of the swine producers indicated they had attended at least one eradication program while about 16% indicated they have not attended any eradication program. In terms of gender, most of the producers were males, with approximately 64% being males and approximately 35% females. Concerning farm size, the responses were regrouped into three categories: small (0–499 hogs), medium (500 to 2499 hogs), and high (above 2500 hogs). Overall, most of the producers were in the small category. We asked about separate and distinct properties where they keep their herd. The mean age of the producers is 36 years, ranging from 20 to 60 years. The average length of time in the current occupation is approximately 9 years, with a minimum of 0 and a maximum of 32 years. We observed a minimum of 0 because some producers may be quite new to the occupation and may have only spent some months in the present occupation.

**Figure 1:**
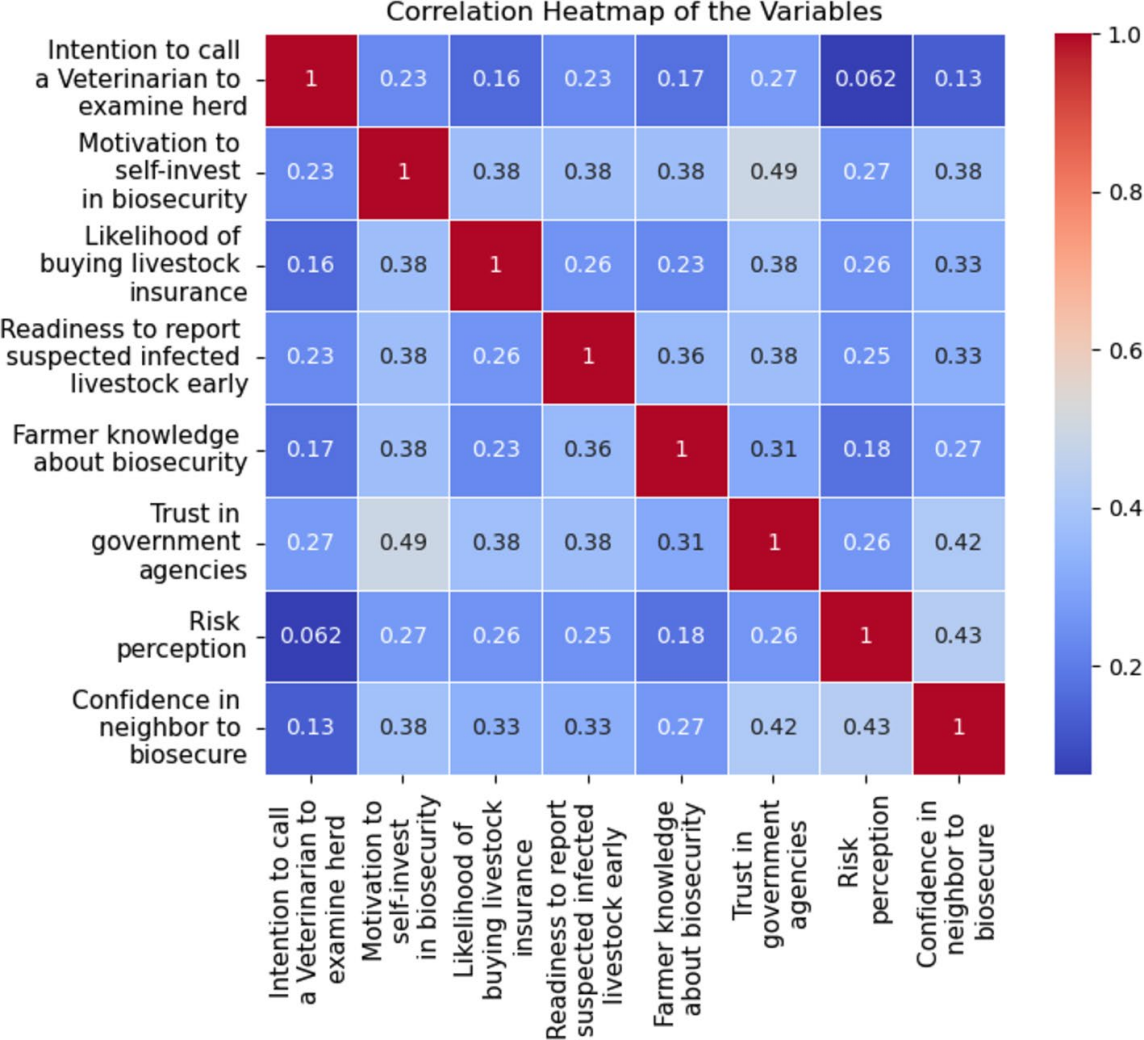
Correlation heatmap of the manifest variables.

The correlation heatmap in Fig. 1 shows low correlation among the manifest variables to be used in the model. The absence of strong correlations between these variables provides prima-facie evidence of homogeneity within the classes, indicating that each class contributes independently to the identification of the classes in the model. Low correlation coefficients satisfy several assumptions of latent class analysis including local independence, homogeneity, and conditional independence.

**Figure 2:**
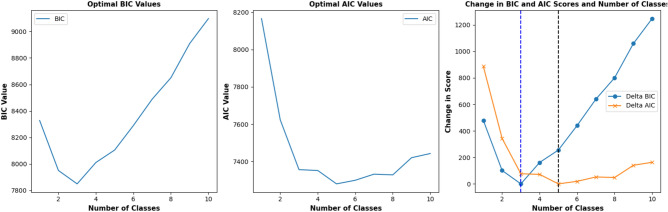
Grid search for the optimal BIC and AIC classes.

To determine the optimal number of classes, we implemented a grid search. The search identified three classes using the Bayesian information criterion (BIC), while the Akaike information criterion (AIC) produced five as the optimal number of classes as shown in Table 2. To further validate three as the optimal number of classes, we implemented the bootstrapped likelihood ratio test (BLRT) following Nylund et al.^39^. The BLRT also gave empirical evidence that supported a 3-class model over a 5-class model. Given the above criteria, the 3-class model was selected to fit the data. Besides, using three classes allows for a parsimonious class selection, ensuring each item has an equal chance of belonging to a particular class. Moreover, the parsimony of the model facilitates a more meaningful interpretation of the results. Any number of classes above this threshold may lead to an over classification problem^40,41^. Figure 2 plots the various BIC and AIC values used in determining the optimal classes in each case.

**Table 2:** Fit indices of latent class analysis of biosecurity adoption attitudes of swine producers.

Number of classes	Log likelihood	AIC	BIC
LC2—2 classes	− 3730.4855	7622.97	7950.62
LC3—3 classes	− 3555.8945	7355.79	7849.28*
LC4—4 classes	− 3512.7113	7351.42	8010.76
LC5—5 classes	− 3435.7547	7279.51*	8104.69

*Note* The figures in with * represent the optimal class model chosen by the AIC and BIC criteria.

### Fitting the model

To further address the misclassification problem, we implement a two-step bias-adjusted model. We followed Vermunt^42^ and applied a maximum likelihood correction (*ml*) to address the downward bias problem. Additionally, we employed a "modal" assignment to ensure each observation is assigned to its most probable latent class Ogurtsov et al.^43^, and we included the soft assignment option to allow individuals to have partial membership in multiple latent classes^43,44^.

**Figure 3:**
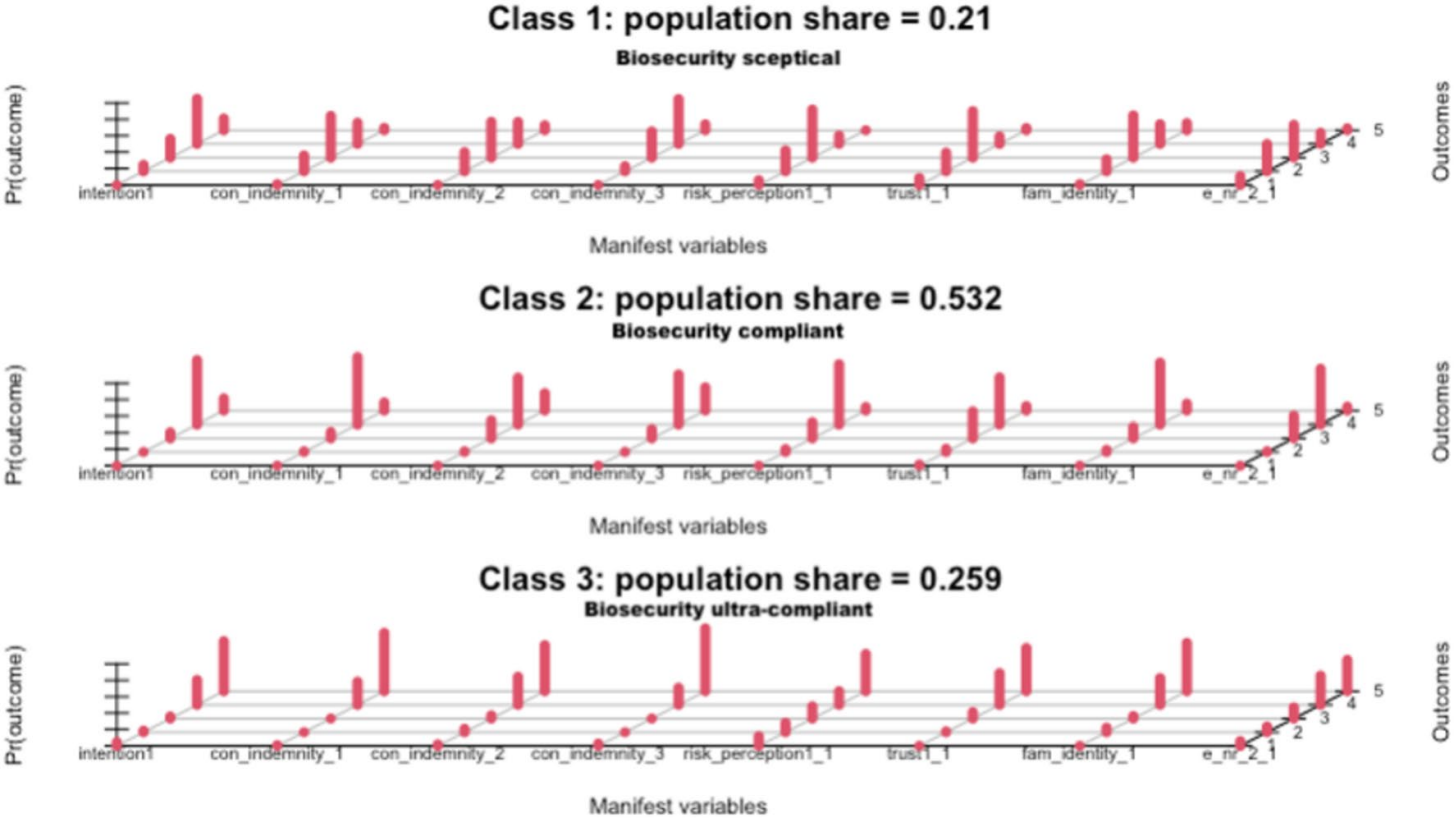
Basic three class latent class model. *Note* Estimation of the three-class basic latent class model using the survey data. Each group of red bars represents the conditional probabilities of the responses. Taller bars correspond dominant conditional probabilities. Intention1 indicates intention to call a veterinarian to examine their herd, con_indemnity1 reflects Motivation to self-invest in biosecurity, con_indemnity1 represents Likelihood of buying livestock insurance, con_indemnity1 signifies Readiness to report disease, fid represents Farmer knowledge about biosecurity, trust1_1 indicates Trust in government agencies, risk_perception1_1 shows risk perception, and e_nr_2_1 reflects Confidence in neighbor to biosecure.

By assigning estimated probability values to each response level for the variables included in the model, we can plot the pattern of each class (see Supplementary Materials for details). Our analysis identified three latent classes (Class 1, Class 2, and Class 3), and the item probabilities were provided. These probabilities represent the conditional likelihood of observing each outcome within each latent class. The class weights represent the proportion of swine producers belonging to each latent class. Specifically, Class 1 comprises 21% of the swine producers who responded to the survey, Class 2 comprises about 53% of the respondents, and Class 3 comprises 26% of the producers who responded to the survey. To visualize this, we plot the conditional probabilities of the three-class basic latent class model in Fig. 3. Note that the difference between the estimated class population shares (21%, 53%, and 26%) and the predicted class membership (by modal posterior probability) (20%, 54%, 26%) are qualitatively similar. These weights indicate the distribution of the latent classes within the dataset, with Class 3 being the most prevalent. The variance among them is 0.6278, indicating a moderate level of homogeneity and precision.

The measurement model parameters (Table 3) show the estimated probabilities of each variable on the Likert scale for each of the three latent classes. These conditional probabilities reveal patterns in the responses. For instance, Class 3 has a higher proportion (38%) of respondents who strongly agree that their neighbors will biosecure, while Class 1 has a higher proportion (33%) of respondents who disagree. Class 2 has a higher proportion of respondents who agree to being confident that their neighbors will biosecure. A high-level overview of the patterns from the conditional item response probabilities reveals that each class has distinct characteristics. For instance, Class 1 producers showed higher rates of unsure responses and low levels of agreement or strong agreement for most variables. We label them 'biosecurity skeptics.' Among Class 2 producers, most agree with responses for most variables and have low levels of disagreement. We label them 'biosecurity compliant.' Class 3 producers tend to strongly agree with most variables and trust government agencies to effectively manage outbreaks. We label them 'biosecurity ultra-compliant.

**Table 3:** Item response probabilities of biosecurity attitudes of swine producers and their class membership.

Variables	Responses	Class 1	Class 2	Class 3
Confidence in neighbor to biosecure	Strongly disagree	0.1073	0	0.0583
	Disagree	0.3271	0.009	0.0694
	Unsure	0.4013	0.2692	0.1359
	Agree	0.1407	0.6755	0.3545
	Strongly agree	0.0236	0.0463	0.3818
Farmer knowledge about biosecurity	Strongly disagree	0.0111	0	0
	Disagree	0.1443	0.0268	0.0555
	Unsure	0.5102	0.1379	0.0304
	Agree	0.2472	0.7498	0.3251
	Strongly agree	0.0872	0.0855	0.5891
Motivation to self-invest in biosecurity	Strongly disagree	0.0111	0	0.0092
	Disagree	0.1899	0.0086	0
	Unsure	0.5079	0.073	0
	Agree	0.2612	0.8197	0.2804
	Strongly agree	0.0299	0.0987	0.7104
Likelihood of buying livestock insurance	Strongly disagree	0	0	0.0184
	Disagree	0.2298	0.0083	0.0411
	Unsure	0.434	0.2137	0.0389
	Agree	0.274	0.5658	0.3404
	Strongly agree	0.0622	0.2122	0.5612
Readiness to report suspected infections	Strongly disagree	0	0	0.0276
	Disagree	0.0667	0	0
	Unsure	0.3151	0.1104	0
	Agree	0.5448	0.6063	0.2079
	Strongly agree	0.0734	0.2833	0.7646
Intention to call a vet to examine herd	Strongly disagree	0	0	0.046
	Disagree	0.0778	0	0.0184
	Unsure	0.231	0.0698	0.0241
	Agree	0.5488	0.7831	0.3015
	Strongly agree	0.1425	0.1471	0.61
Risk perception	Strongly disagree	0.0578	0	0.1176
	Disagree	0.2506	0.0357	0.1147
	Unsure	0.5831	0.1895	0.1491
	Agree	0.1085	0.7315	0.165
	Strongly agree	0	0.0433	0.4536
Trust in government agencies to manage outbreak	Strongly disagree	0.0854	0.0148	0
	Disagree	0.2258	0.0387	0.0096
	Unsure	0.5678	0.3199	0.078
	Agree	0.0935	0.5718	0.3856
	Strongly agree	0.0276	0.0547	0.5268

*Note* This table provides item response probabilities for various biosecurity attitudes among swine producers categorized into three classes (1, 2, and 3). The probabilities indicate the likelihood of respondents choosing each response category (Strongly Disagree, Disagree, Unsure, Agree, Strongly Agree) for each outcome variable.

We note specific observations across the classes. For instance, concerning the confidence in their neighbors to biosecure, we observe that the biosecurity compliant group has the highest agreement, with about 68% of the respondents agreeing and 5% of them strongly agreeing. Meanwhile, the biosecurity ultra-compliant group strongly agree to having confidence in their neighbors to biosecure. Moving on to farmers' knowledge about biosecurity, we observe that about 59% of the biosecurity ultra-compliant producers strongly agree that they have knowledge about biosecurity, followed by about 75% of the biosecurity compliant producers who agree to having knowledge about biosecurity. Regarding the likelihood of buying livestock insurance, we observe that 56% of the producers who strongly agree were in the biosecurity ultra-compliant group, while about 57% of the producers who agree to buying livestock insurance were in the biosecurity compliant group. In the biosecurity sceptics group, about 43% of the producers disagreed to the likelihood of buying livestock insurance. Motivation to self-invest in biosecurity predominantly shows that about 50% of the respondents who are unsure belong to the biosecurity sceptics group, while about 82% of the respondents who strongly agree to self-investing in biosecurity belonged to the biosecurity compliant group. The biosecurity ultra-compliant producers have about 71% of them agreeing to self-invest in biosecurity.

Readiness to report suspected infections on farms varied among groups: 55% of biosecurity sceptics agreed, 60% of biosecurity compliant producers agreed, and 77% of biosecurity ultra-compliant producers strongly agreed. While readiness to report infections is related to the intention to call a veterinarian, the data shows that 55% of biosecurity sceptics and 61% of biosecurity compliant producers agreed to call a veterinarian to examine their herd in the event of an infection, while 77% of biosecurity ultra-compliant producers strongly agreed to call a veterinarian. This suggests a strong positive relationship between readiness to report infections and intention to seek veterinary care.

To further assess the pattern of behavior among the producers, we observe that about 58% of the producers in the biosecurity sceptics group were unsure about the risk of ASF outbreak on their operations, while 73% of the producers in the biosecurity compliant group agreed that their production operations were at risk of ASF. About 45% of the producers in the biosecurity ultra-compliant group strongly agreed that their production operations were at risk of ASF. Finally, we also observe that about 57% of the producers in the Biosecurity sceptics group were unsure about their level of trust in the government to effectively manage a disease outbreak. In contrast, about 57% of the producers in the biosecurity compliant group agreed that they trust the government agencies to effectively manage an ASF outbreak in the US. The biosecurity ultra-compliant producers had about 53% of them strongly agreeing that they trust government agencies to effectively manage outbreaks.

All in all, given that the biosecurity sceptics had lower confidence in their neighbors to biosecure, most of them were unsure about their likelihood of buying livestock insurance, and had a low level of trust in the government to effectively manage an outbreak, we label this group the "biosecurity sceptics". The biosecurity compliant producers, on the other hand, showed moderate to high motivation to self-invest in biosecurity, a willingness to buy livestock insurance, and greater knowledge about biosecurity. The biosecurity ultra-compliant producers also showed the strongest motivation to self-invest in biosecurity and higher levels of agreement in all areas related to biosecurity.

### Test of difference across the classes

After identifying the class membership for the selected variables, we conduct a one-way ANOVA test to check if there are any significant differences across the three classes. Table 4 reports the output of the ANOVA (Welch’s) test with a p value less than one percent. Our test confirms significant differences across the three classes. With these results, we further conduct a post hoc analysis to observe where those differences lie. The results presented in Table 5 show that there is a 95% chance that the classes are significantly different from each other. We demonstrate this by comparing results between classes for each variable.

**Table 4:** One-way ANOVA test of differences.

Variables	F value
Intention to call a veterinarian to examine herd	23.1406***
Motivation to self-invest in biosecurity	203.0206***
Farmer knowledge about biosecurity	77.7252***
Likelihood of buying livestock insurance	65.2826***
Readiness to report suspected infected livestock	62.1917***
Trust in government agencies	120.8024***
Risk perception	46.4250***
Confidence in neighbors to biosecure	75.5754***

*Note* ***denotes significance level at 1%.

**Table 5:** Post-Hoc analysis: pairwise comparison of group means.

Comparison	Group1	Group2	Mean difference
	Class 1	Class 2	0.346***
Intention to call a veterinarian to examine herd	Class 1	Class 3	0.688***
	Class 2	Class 3	0.342***
	Class 1	Class 2	0.9346***
Motivation to self-invest in biosecurity	Class 1	Class 3	1.6312***
	Class 2	Class 3	0.6966***
	Class 1	Class 2	0.7106***
Farmer knowledge about biosecurity	Class 1	Class 3	1.2509***
	Class 2	Class 3	0.5403***
	Class 1	Class 2	0.8343***
Likelihood of buying livestock insurance	Class 1	Class 3	1.2623***
	Class 2	Class 3	0.4281***
	Class 1	Class 2	0.570***
Readiness to report suspected infected livestock	Class 1	Class 3	1.0776***
	Class 2	Class 3	0.5076***
	Class 1	Class 2	0.8264***
Trust in government agencies	Class 1	Class 3	1.6718***
	Class 2	Class 3	0.8454***
	Class 1	Class 2	1.0636***
Risk perception	Class 1	Class 3	1.0197***
	Class 2	Class 3	-0.044
	Class 1	Class 2	1.1508***
Confidence in neighbors to biosecure	Class 1	Class 3	1.3299***
	Class 2	Class 3	0.1791

*Note* The table presents the results of post-hoc pairwise comparisons of group mean for various variables related to biosecurity attitudes and behaviors among different classes of producers (Class 1, Class 2, and Class 3). Mean differences between groups are reported, with statistically significant differences denoted by asterisks (***p < 0.001). The comparisons indicate significant differences in intention to call a veterinarian, motivation to self-invest in biosecurity, farmer knowledge about biosecurity, likelihood of buying livestock insurance, readiness to report suspected infected livestock, trust in government agencies, risk perception, and confidence in neighbors to implement biosecurity measures.

Table 5 shows the multiple comparison of means using the Tukey HSD with a family-wise error rate of 5%. Comparing the groups across the classes for all the variables, the test reported significant mean difference between the classes. For instance, when comparing the intention call a veterinarian to examine their herd, The results show that Class 2 has a significantly different intention to call a veterinarian as compared to Class 1, with a difference of 0.346. Implying that the intention to call a veterinarian to examine their herd is higher in class 1 compared to class 2. Similarly, with a mean difference of 0.688, the intention to call a veterinarian to examine their herd is 0.688 units higher in class 1 compared to class 3. The mean difference between class 2 and class 3 is 0.342, implying that intention to call a veterinarian to examine their herd is 0.342 units higher for producers in class 2 compared to producers in class 3.

Table 6 presents the results of our latent class regression analysis. Note that this estimation uses a generalized multinomial logit link function, with the reference category constrained to 1. The selected covariates in the model—gender of the producers, participation in at least one eradication program, and the share of income derived from their production operations—have a statistically significant effect on the biosecurity attitudes of the producers. Notably, income had the most significant effect on the biosecurity attitudes of the producers, followed by gender and eradication program.

**Table 6:** Regression results for 3 latent classes.

Predictors	Biosecurity compliant vs. biosecurity sceptics		Biosecurity ultra-compliant vs biosecurity sceptics	
	Coefficient	Standard error	Coefficient	Standard error
Size of farm	− 0.38811	0.42044	− 0.86518**	0.3979
Gender of farmer (male)	− 0.83426**	0.38822	0.59218	0.47883
Attending at least one eradication program	0.91594*	0.50106	− 0.20513	0.52645
Share of income derived from production operation	0.47098**	0.22099	0.60435**	0.26775
Length of time in current occupation	− 0.04332	0.03559	0.02925	0.03243

*Note* This table presents regression results comparing predictors between Biosecurity Compliant and Biosecurity Sceptics, as well as Biosecurity Ultra-Compliant and Biosecurity Sceptics. Coefficients and standard errors are provided and statistical significance levels (*p < 0.10, **p < 0.05).

## Discussion

As stated in the results, the latent class analysis revealed three classes. The predicted class means of each class in the sample biosecurity sceptics (21%), biosecurity compliant (53%) and biosecurity ultra-compliant (26%)). The biosecurity sceptics tend to respond with uncertainty and disagreement to biosecurity-related questions, while biosecurity compliant producers tend to agree with these questions. biosecurity ultra-compliant producers strongly agree and have positive attitudes towards biosecurity practices. These categories may be associated with producers' beliefs, intentions, and understanding of biosecurity practices on their farms. For example, producers who strongly agree to immediately report suspected infections on their farms, regardless of the current indemnity policy, are likely to belong to the biosecurity ultra-compliant class. In contrast, producers who are unsure about reporting suspected infections are likely to belong to the Biosecurity sceptical class.

Given that the estimated result in Table 6 uses a multinomial framework, we can interpret the coefficients in Table 6 as the log-ratio prior probability. The share of income from production operations was significant in all categories. The results indicate that the probability of belonging to the biosecurity compliant class for producers who derive a larger share of their income from their production operations is 0.615. With low income as the reference category, the log odds of producers with moderate- and high-income shares would increase by 0.471 for the biosecurity complaint group than the sceptical biosecurity group. This suggests that moving from low to a higher income group would increase the log-odds of belonging to the biosecurity compliant group. Similarly, the results from the share of income derived from production operations for those in the biosecurity ultra-compliant group show a positive coefficient, indicating that as the share of income derived from production operations increases, the log-odds of belonging to the biosecurity ultra-compliant class also increases. Thus, producers with a larger share of their income coming from their production operations are more likely to belong to the biosecurity ultra-compliant class, reflected in the predicted probability of 0.646. This is consistent with the findings of Otieno et al.^30^, who observed that high biosecurity adopters were positively associated with high on-farm income.

Moving on to the comparison of the biosecurity ultra-compliant class and the biosecurity sceptical class, in terms of production size. The coefficient of production size indicates that the probability of belonging to the biosecurity ultra-compliant class for producers with large farms is 0.296. This suggests that the size of a producer's farm has a significant impact on the likelihood of them belonging to the biosecurity ultra-compliant class. Specifically, the result indicates that producers with larger farms have a 0.296 probability of being classified as biosecurity ultra-compliant. Conversely, this implies that small-scale producers are less likely to be ultra-compliant in terms of biosecurity practices. Given that small scale producers are less likely to belong to the biosecurity ultra-compliant group, policy directions should focus on providing targeted support such as low interest loans, subsidies, or grants to access resources that will enable them to adopt biosecurity practices. Some other policies could include certification programs or market access rewards. Our finding is consistent with Schembri et al.^45^, who observed that herd size impacts biosecurity effectiveness and that biosecurity implemented by small-scale producers was mostly inadequate.

The coefficient of the gender of the producers (− 0.858) indicates that being female decreases the log-odds of belonging to the biosecurity compliant class compared to the biosecurity sceptical class. Transforming this coefficient into a predicted prior probability, we find that the probability of belonging to the biosecurity compliant group when the gender of the producer is female is 0.304. This suggests that being female decreases the likelihood of belonging to the biosecurity compliant class. Policies should seek promote gender-sensitive extension services to women while empowering women’s role in agriculture to while providing resources to enhance their decision making. This finding contrasts with Weber et al.^46^ that women exhibit greater risk aversion or risk neutrality in the context of biosecurity compared to men.

Attending at least one eradication program significantly increases the likelihood of being in the biosecurity compliant class. With a 10 percent level of significance, the coefficient of 0.91594 suggests that such attendance increases the log-odds of belonging to the biosecurity compliant class. Using the logistic function, the predicted probability of belonging to the biosecurity compliant class for those who attended at least one eradication program is about 0.714. One can glean from this finding that increasing investment in eradication programs could convert the 21% of skeptics into compliant adopters. Our finding is consistent with Baye et al.^18^ and Devitt et al.^47^, who demonstrated that attending at least one eradication program could influence biosecurity adoption. On the other hand, one can also argue that interest in biosecurity may also drive attendance to these eradication programs. Although we did not explore this relationship, future studies may investigate this reverse causal relationship. We argue that by attending these eradication programs, producers become more informed about the consequences of these diseases on food security and the importance of compliance with national and international animal health regulations. Baye et al.^18^ and Devitt et al.^47^, who demonstrated that attending at least one eradication program could influence biosecurity adoption. Moreover, this has implications for food policy as more investment in eradication programs could convert the 21% sceptics into compliant adopters.

## Conclusion

In conclusion, our latent class analysis and latent class regression revealed significant associations between producer characteristics and biosecurity attitudes. We identified distinct attitudes and behaviors towards biosecurity practices among producers and examined the relationship between producers' characteristics and class membership. These classes are the biosecurity sceptics, the biosecurity compliant, and the biosecurity ultra-compliant. Our findings show that producers belonging to the ultra-compliant group includes higher income producers and those participating in eradication programs. The sceptical group were associated with lower income and smaller scaled farms. Our results highlight that small scale producers are less likely to belong to the ultra-compliant group, likely due to resource constraints.

This study contributes to the development of evidence-based policies and interventions aimed at improving biosecurity practices and protecting animal and public health. We find positive effect of eradication programs in improving producer attitudes towards biosecurity. Food policy makers may consider additional investments in eradication programs targeted at the 21% biosecurity sceptics discovered in this empirical. Moreover, we also observe that producers with a higher share of their income from their production operation and large-scale producers are more likely to invest in biosecurity practices than small scale producers and livestock producers who generate a lower share of their income from their production operations. Several policy support systems can be gleaned from this finding. For instance, we argue that targeted support towards small scale producers like market entry assistance and certification can enhance their biosecurity compliance. These interventions together with targeted interventions, such as risk communication, training programs and monitoring, can all contribute to enhance biosecurity adoption among producers. For instance, Merrill et al.,^6^ show how graphical risk communication lead to an improvement in biosecurity compliance in an experimental game while Racicot et al.,^48^ show that training together with effective monitoring lead to increased biosecurity adoption about fowl producers in Canada.

By understanding the factors that influence biosecurity attitudes, we can develop effective strategies to promote proactive risk management and mitigate the risk of TAD impacting global food security. Ultimately, these results provide valuable insights into factors influencing biosecurity attitudes and behavior, particularly in major swine production states. A limitation of our study is that we did not account for different production types (Farrow to Finish, Farrow to Feeder, and Feeder to Finish) and the regional or cultural differences across states in our analysis. This could have potentially lead to the unexplained similarities in attributes for the classes. Considering the nature of the swine industry, this study would greatly benefit from understanding the different contractual relationships between swine owners and producers. Future studies must also consider panel data to estimate changes in the attitudes of livestock producers over time under different market and policy/governance regimes.

## Methods

As a confirmation, all methods were carried out in accordance with the relevant guidelines and regulations by the Institutional review board at the University of Vermont. The study was approved under IRB number STUDY00001612. We also confirm that all the experimental protocols were approved by the Institutional Review Board at the University of Vermont. Before launching the survey, we sought informed consent from the respondents before they responded to the survey. Participants were free to opt out from the survey at any point in time without any cohesion.

The survey was developed in Qualtrics, and data was collected from March to August 2022. Prior to the full launch of the survey, we piloted the survey with a sample of 10 producers from Vermont. Owing to data privacy concerns from various state swine producer associations, we were unable to obtain a list of their members for the survey design. As such, we relied on the directors of the various swine producer associations including the American Association of Swine Veterinarians (AASV), the National Pork Board (NPB), and extension directors across the US to help distribute the survey to swine producers whom they had contact. They elected to place the survey on their listservs and newsletters for their members. To reduce the uncertainty and bias resulting from this sampling technique, we ensured that the survey was distributed to these institutional heads at different time frames. This ensured that members of the various swine producer groups had equal chance of responding to the survey at their own convenience.

The combination of convenience and snowball sampling designs enabled us to gather more data than the anticipated sample size. To ascertain the sample size, Kelley^49^ and Kelley et al.^50^ developed the MBESS package in R for computing the sample size. Our resulting estimate, after applying this method to approximately 60,000 swine producers, yielded 369 respondents with a 5% margin of error and a 95% confidence interval. Although our study had no control over the sampling frame, about 729 respondents responded to the survey, indicating that more than 62% of the expected respondents attempted to respond to the survey. Respondents in the study were from 49 states excluding Rhode Island. Moreover, we suspected the presence of multiple responses; therefore, duplicates were identified by their unique IDs and subsequently removed from the dataset. We also dropped incomplete responses. These responses include swine producers who either only opened the survey or responded to the first two questions before discontinuing. After cleaning the data, our resulting sample size was 422 fully completed survey.

With the majority of the responses coming from pork-producing regions in the west, south, and northeast of the US. Surprisingly, the fewest responses came from major pork-producing states such as Iowa, Illinois, Minnesota, and Indiana, which are located in the corn belt regions. We attribute this to both a "distribution bias" and a "seasonal bias" during our data collection process. Studies by Pennings et al.^51^ has shown that the optimal time for distributing surveys to producers in the Midwest is usually between January and February, while the least favorable time to respond is between March and November. This may explain the low response rate from our survey in this region.

The design of the survey included questions about the demographic and operations characteristics of producers. We also collected information on risk perception, trust in government agencies to effectively manage outbreaks, motivation to self-invest in biosecurity, willingness to buy livestock insurance, and readiness to report infections on farms. As a verification check, we randomly sampled 20 respondents and called them to verify if they were swine producers. All 20 respondents mentioned they were involved in swine production.

Our survey aimed to identify behavioral attitudes and biosecurity decision making strategies of livestock producers through an understanding of their risk perceptions, trust in government agencies, biosecurity knowledge of livestock making of swine producers through their intentions to act in response to ASF. The survey included questions about farmers motivation to self-invest in biosecurity, likelihood of purchasing livestock insurance and readiness to report diseases. The survey consisted of questions based on three scenarios together with demographic and operational characteristics. A pre-amble to the first scenario is presented as follows:

“*Imagine African swine fever is likely to be detected in your location. Herds testing positive for African swine fever, as well as their neighboring herds, will be euthanized in order to control the spread of the disease. Typically, all susceptible animals including pigs and boars may be euthanized. The clinical signs of African swine fever are high fever, weakness and difficulty in standing, vomiting, diarrhea, coughing, miscarriage, and red or blue blotches around the ears and snout. It is brought to your attention that many of the swine in your herd appear weak and have difficulty standing. Several of the animals are noticeably lame. Some of the animals appear to be vomiting and coughing”.*

Based on this scenario, producers were asked to respond to a series of statements to assess their intentions and attitudes. They rated their responses on a 5-point Likert scale from "Strongly Disagree" to "Strongly Agree". For instance, we asked about their intention: "Please indicate how strongly you agree with the following statement: 'In Situation #1, I would ask a veterinarian to examine my herd'."

The second scenario considers a potential change in the current policy for indemnification. We presented the scenario as follows: "As a strategy to contain and prevent the spread of the African swine fever disease and be eligible for compensation for losses, you can implement a biosecurity plan. This plan may include investing in setting up a disinfecting area, keeping an isolation area and a line of separation, having a biosecurity manager, and proper waste and carcass disposal." From this scenario, producers were presented with conditional statements about their willingness to self-invest in biosecurity to be eligible for indemnity, likelihood of purchasing livestock insurance, and readiness to report any suspected infections on their farms. For instance, to reflect their motivation to self-invest in biosecurity practices, we asked: "If funds were available from the government but were contingent on showing your biosecurity plans, it would motivate me to invest and show my biosecurity plans." To examine the likelihood of buying livestock insurance, we asked: "If funds were available from the government but were contingent on showing your biosecurity plans, it would encourage me to invest some percentage of my production operations in private farm insurance." And to examine readiness to immediately report suspected infected livestock on their farms, we asked: "If funds were available from the government but were contingent on showing your biosecurity plans, it would encourage me to report suspected infected livestock early."

In the final part of the survey, we asked questions pertaining to the demographic and operational characteristics of the producers. This included questions about their age, gender, educational level, number of years involved in the industry and in swine operations, share of income, proximity to their farms, the size of their herd, their involvement in any eradication programs, the number of separate and distinct properties where their pigs are kept, and their location (state).

### Empirical framework

To capture producers' unobserved heterogeneity, we rely on latent class analysis to identify hidden subpopulations to which different swine producers may have similarities in preferences. Apart from its suitability for categorical data, latent class analysis is deemed superior to other clustering techniques because one can determine the fitness of the model. Latent class analysis also provides enhanced estimation accuracy compared to alternative methods such as k-mode clustering and hierarchical clustering^52^ (McCutcheon and Pekarik, 2014). According to^41,53^, latent class analysis, either used alone or in conjunction with factor analysis, offers distinct advantages in evaluating specific unobserved underlying subpopulations.

To start with, assume that the response of a livestock producer *i* on an item *n* is denoted by *Y*_*in*_. Where Y is the response vector. Note that these variables are categorical and even though they are ordinal in nature, they are treated as nominal. Mathematically, the function defining the latent class is shown in Eq. (1):

1 $$P({Y}_{i})=\sum_{\vartheta =1}^{\vartheta }P({X}{^\prime}=\theta )P({Y}_{i}|{X}{^\prime}=\theta )$$

where ${X}{\prime}$ is a vector of the manifest variables and $\vartheta$ is the latent class of $\theta$ classes. To estimate the probability of each livestock producer belonging to a particular class assuming local independence. Given *Y*_*in*_, we can estimate the posterior probability that each producer belongs to each class, conditional on the observed values of the manifest variables. Using bayes rule, we can estimate the probability of a livestock producer *i* choosing alternative *n* in a given set of responses conditioned on belonging to class $\vartheta$. For example, we can examine the probability of having a high level of trust in government agencies to manage disease outbreaks, conditional on belonging to class $\vartheta$. Expressly, we can write the posterior probability as shown in Eq. (2):

2 $$P({Y}_{in }= j| class = \vartheta ) = exp({X}_{inj}{\prime}{\beta }_{\vartheta }){({\sum }_{j=1}^{j}exp({X}_{inj}{\prime}{\beta }_{\vartheta }))}^{-1}$$

where ${\beta }_{\vartheta }$ is the class specific parameters signifying homogeneity within each latent class.

In our model estimation, we fitted a 2-step latent class model with 122 estimated parameters and 422 observations. To determine an appropriate class size, several approaches have been suggested, including entropy, log likelihood, and information criteria (Akaike information criterion (AIC) and Bayes information criterion (BIC)). According to Beath^54^, selecting the most parsimonious class is ideal, and they recommend using the BIC because it considers the number of observations and selects the model with fewer classes^39^. We implemented a grid search for the optimal number of classes for the model.

After identifying the three classes we estimated a latent class regression by including covariate to predict the producers’ latent class membership. We implement the so-called one-step technique using the poLCA package in R^38^. Unlike the so-called “three step model” where one would have to estimate the basic latent class model, calculate the predicted posterior class membership probabilities and them using them as dependent variables in the multinomial regression with the desired covariates, we opt for the one-step approach because the coefficients on the covariates are estimated simultaneously as part of the latent class model. More so, the approach uses the Newton Raphson expectation minimization to maximize the function for the calculation of the posterior probabilities^38^.

Together with a set of covariates, we estimate a one-step latent class regression to understand how a set of demographic and operational characteristics of the producer will predict membership to each class. The log odds for the model for producer *i* in class *k* is given in Eq. (3):

3 $${logit(P}_{ik}) = {\beta }_{0k}+ {\beta }_{1k}{Covariats}_{i}$$

Using the logistic function, we can transform the log-odds into probabilities. Hence the probability of each class membership is shown in Eq. (4):

4 $${p}_{ik}=\frac{{exp}^{{logit(p}_{ik})}}{\sum_{j}{exp}^{logit({p}_{ij})}}$$

### Supplementary Information

Supplementary Information.

## Data Availability

The dataset and analysis have been published under this institutional repository. Find the link here. Baye, R. (2024). Replication data for: "A latent class analysis of biosecurity attitudes and decision-making strategies of swine producers in the United States" [Data set]. Harvard Dataverse. Data
