# Supplementary material for: A latent class analysis of biosecurity attitudes and decision-making strategies of swine producers in the United States
Source: Sci Rep. 2024 Aug 5;14:17427. doi: 10.1038/s41598-024-67385-z (PMC11300889; doi:10.1038/s41598-024-67385-z)
Supplement: Supplementary file 1 — Supplementary Information. [file 41598_2024_67385_MOESM1_ESM.docx]

**Supplementary materials.**

**Copy of the survey**

**Imagine yourself in this situation.**

**Imagine yourself in this situation**

**Scenario # 1**

Imagine African swine fever is likely to be detected in your location. Herds testing positive for African swine fever, as well as their neighboring herds, will be euthanized in order to control the spread of the disease. Typically, all susceptible animals including pigs and boars may be euthanized.

The clinical signs of African swine fever are high fever, weakness and difficulty in standing, vomiting, diarrhea, coughing, miscarriage, and red or blue blotches around the ears and snout. It is brought to your attention that many of the swine in your herd appear weak and have difficulty standing. Several of the animals are noticeably lame. Some of the animals appear to be vomiting and coughing.

Based on your own experiences and the demands of your swine operation, please answer the following questions regarding Situation # 1**Please indicate how strongly you agree with the following statements.**

|  | Strongly Disagree | Disagree | Unsure | Agree | Strongly Agree |
| --- | --- | --- | --- | --- | --- |
| 1. In situation 1, I would ask a veterinarian to examine my herd |  |  |  |  |  |
|  |  |  |  |  |  |

This reflects intention to call a veterinarian to examine their herd.


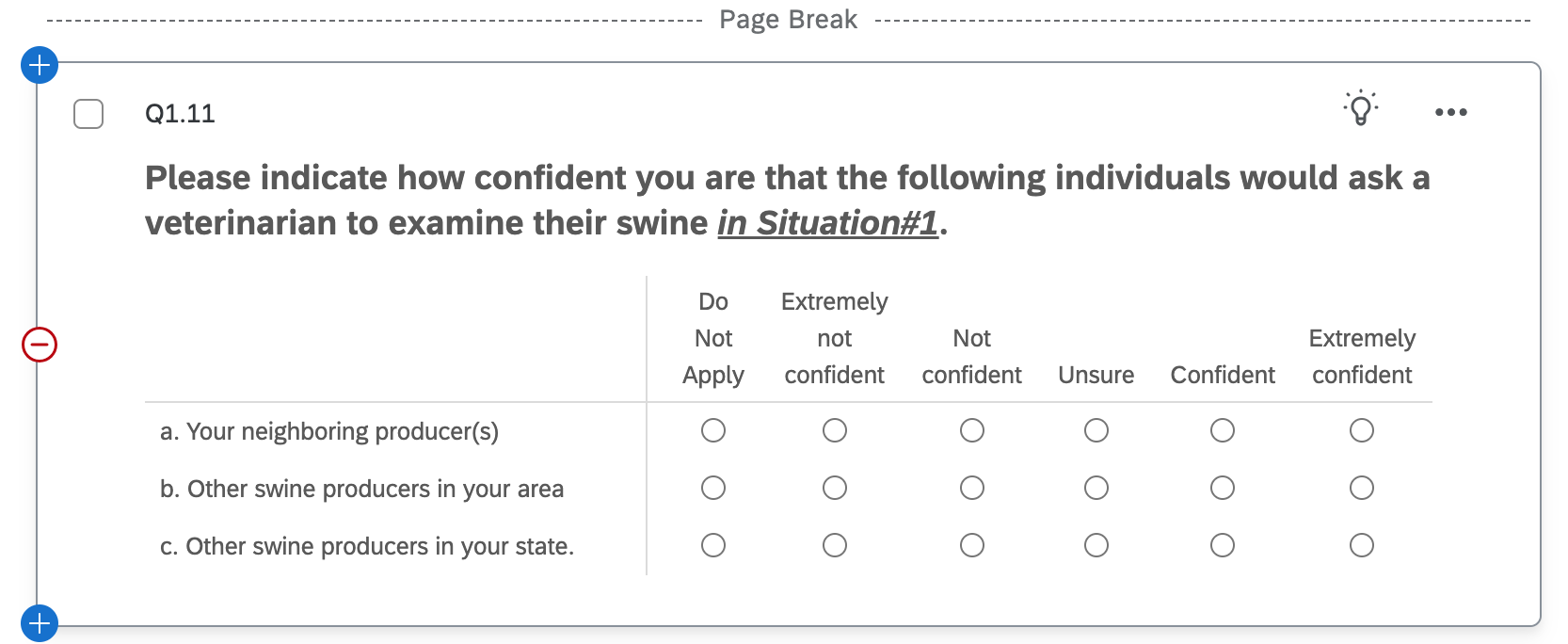


**Scenario 2**

As a strategy to contain and prevent the spread of the African swine fever disease and be eligible for compensation for losses, you can implement a biosecurity plan. This plan may include investment in setting up a disinfecting area, keeping an isolation area and a line of separation, having a biosecurity manager, and proper waste and carcass disposal.

*If funds were available from the government but were contingent on showing your biosecurity plans,*

|  | Strongly Disagree | Disagree | Unsure | Agree | Strongly Agree |
| --- | --- | --- | --- | --- | --- |
| a. It will motivate me to invest and show my biosecurity plans. |  |  |  |  |  |
| b. It will encourage me to invest some percentage of my income in private farm insurance. |  |  |  |  |  |
| c. It will encourage me to immediately report suspected infected livestock . |  |  |  |  |  |

**Farmer knowledge about biosecurity**

Please indicate how desirable or undesirable the following outcomes are for you personally.

|  | Strongly Disagree | Disagree | Unsure | Agree | Strongly Agree |
| --- | --- | --- | --- | --- | --- |
| a. It will motivate me to invest and show my biosecurity plans. |  |  |  |  |  |
| b. It will encourage me to invest some percentage of my income in private farm insurance. |  |  |  |  |  |
| c. It will encourage me to immediately report suspected infected livestock . |  |  |  |  |  |

**Scenario 3**

**Imagine yourself in the following situation.**
After African swine fever is identified in your location, producers ought to restrict the movement of anything that could spread the disease. These movement restrictions may last for many weeks. 
These movements restrictions will cover products (i.e., sausages, bacon, fertilizers) from swine. In addition, the movement of vehicles, including feed trucks, and personnel will also be restricted. 
People, other types of animals, vehicles, and equipment may only be allowed to move following an extensive disinfection process that involves the application of an appropriate chemical disinfectant and a mandatory wait period before coming into contact with susceptible animals.

Given the scenarios above, please indicate how you strongly agree or disagree with the following statement:
I believe that the following agencies would act in my best interest in managing an outbreak of African Swine Fever.

|  | Strongly Disagree | Disagree | Unsure | Agree | Strongly Agree |
| --- | --- | --- | --- | --- | --- |
| 1. United States Department of Agriculture (FEMA) |  |  |  |  |  |
| b.Federal Emergency Management Agency (FEMA). |  |  |  |  |  |
| c. Relevant State Animal Health Entity . |  |  |  |  |  |

Risk perception

**Please indicate how strongly you agree or disagree with the following statements.**

|  | Strongly Disagree | Disagree | Unsure | Agree | Strongly Agree |
| --- | --- | --- | --- | --- | --- |
| a. The risk of an outbreak of African Swine Fever in the USA is very great |  |  |  |  |  |
| b. The risk of an outbreak of African swine fever in my operation is very great.. |  |  |  |  |  |

Famer knowledge about biosecurity

**Please indicate how strongly you agree or disagree with the following statements.**

|  | Strongly Disagree | Disagree | Unsure | Agree | Strongly Agree |
| --- | --- | --- | --- | --- | --- |
| a. Farmers should not allow visitors near their livestock. |  |  |  |  |  |
| b. Farmers should ensure workers and visitors wear clean footwear and clothes during farm visits. |  |  |  |  |  |

f. What is your sex?

Male

Female

Age in years ------------

Length of time in current occupation in years ---------------

Percentage of income derived from swine operations.-----------

Which federal or state eradication program have you been directly involved with

Please indicate your state.

1. Secure Pork Supply program
2. Brucellosis eradication program
3. Porcine Epidemic Diarrhea virus
4. Pseudorabies in swine
5. Other (please specify)

**Figure 4: Heatmap of Conditional item response probabilities of the manifest variables
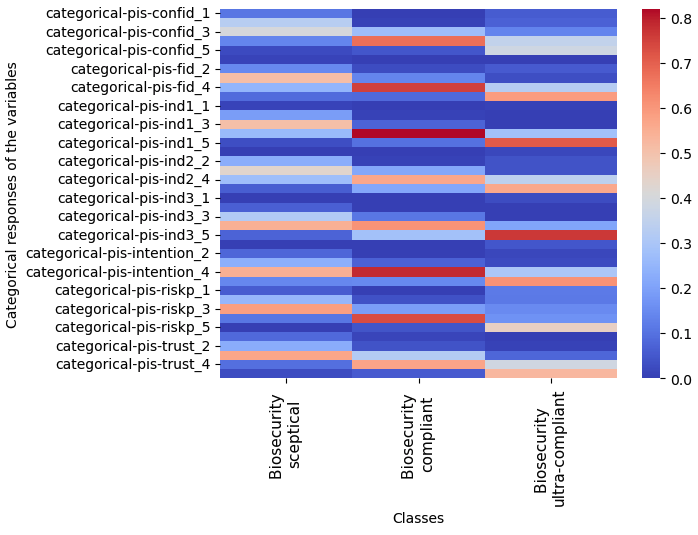
**

**Note:** 1, 2, 3, 4, and 5 represents the categorical responses on a Likert scale. Where 1 is strongly disagree, 2 is disagree, 3 is unsure, 4 is agree and 5 is strongly agree. categorical-pis-confid is confidence in neighbors to biosecure, categorical-pis-fid is farmers knowledge about biosecurity, categorical-pis-ind1 is motivation to self-invest in biosecurity, categorical-pis-ind2 is likelihood of buying livestock insurance, categorical-pis-ind3 is readiness to report suspected infections on farms. categorical-pis-intention is intention to call a veterinarian to examine their herd. categorical-pis-riskp is risk perception of the farmer and categorical-pis-trust is trust in government agencies to effectively manage an outbreak. This was plotted in python

**Table 8: Descriptive statistics of the continues variables in the model**

|  | Count | Mean | Std.dev | Min | Max |
| --- | --- | --- | --- | --- | --- |
| Length of time in current occupation | 414 | 8.650 | 6.117 | 0 | 32 |
| Age | 422 | 35.815 | 8.063 | 20 | 60 |

**Figure 5: Differences in conditional item probabilities across different latent classes.**


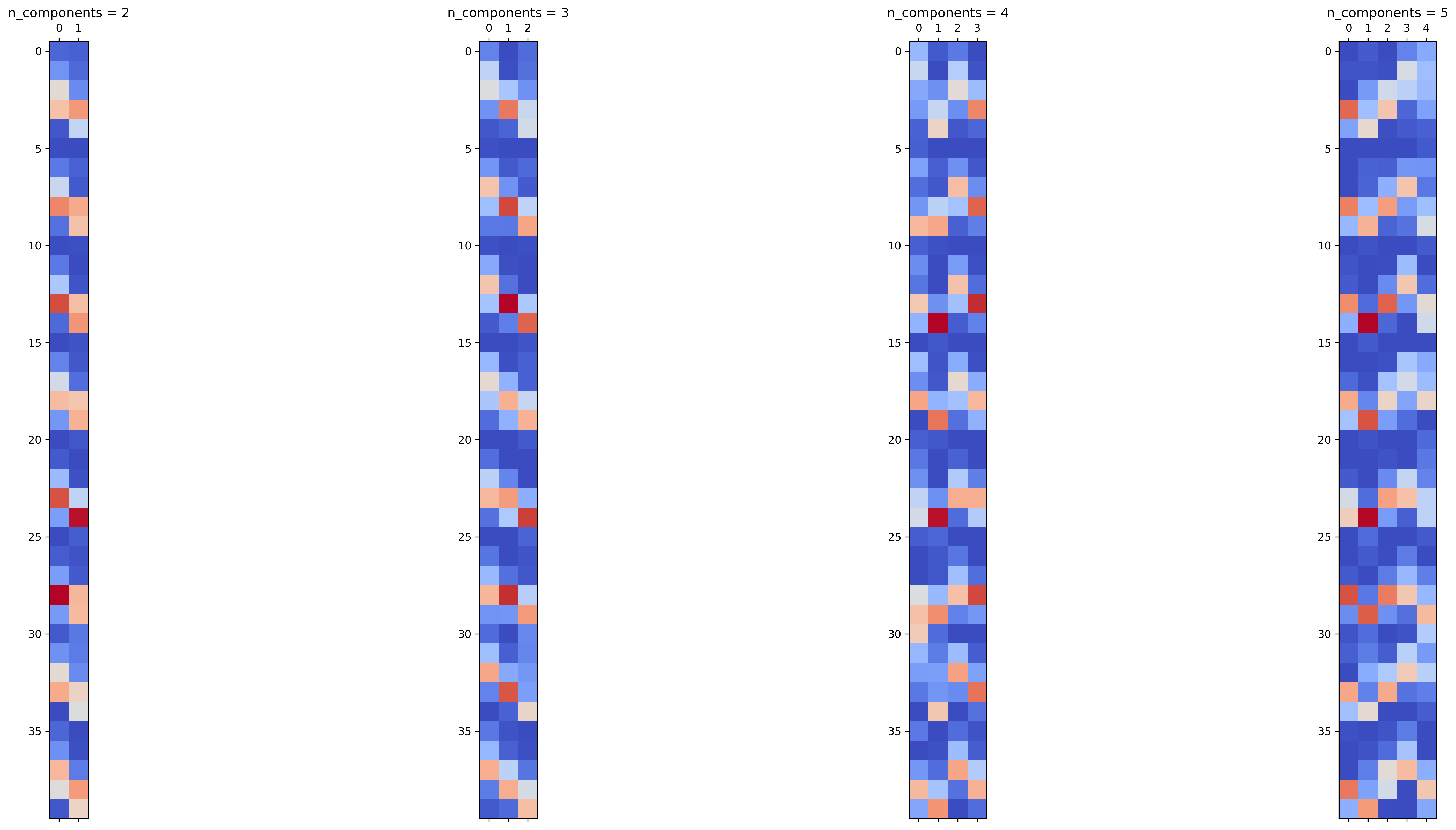


Note: The variables on the Y-axis correspond to the variables used in the mode. In the order (0-35) corresponds to Intention to call a veterinarian to examine herd, Motivation to self-invest in biosecurity, Farmer knowledge about biosecurity, Likelihood of buying livestock insurance, Readiness to report suspected infected livestock, Trust in government agencies, Risk perception, Confidence in neighbors to biosecure. This was plotted in python

**Figure 6: Regional count of total number of responses**


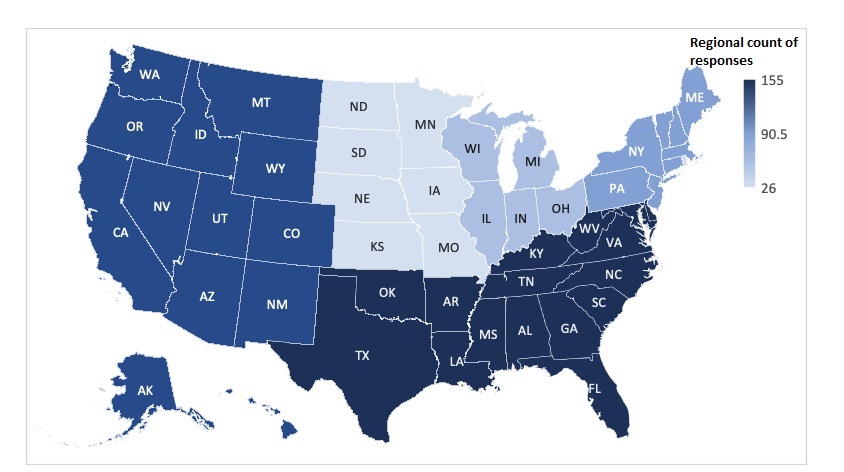


Note: Map was created using Microsoft Excel.
